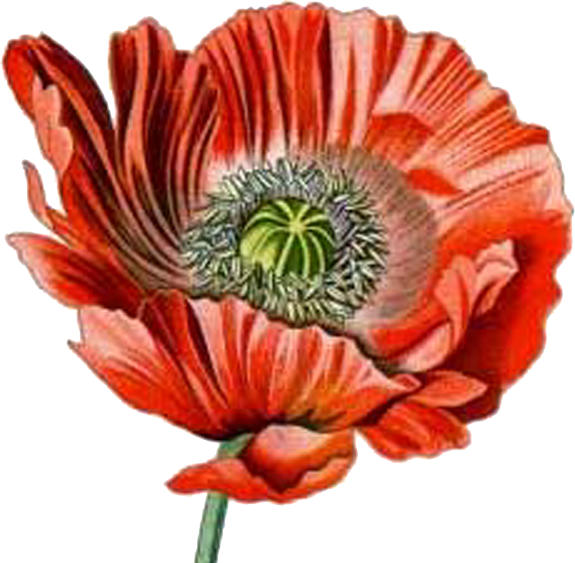# Toward an Individual Approach to Methadone Therapy of Heroin Addicts

**DOI:** 10.1371/journal.pmed.0030128

**Published:** 2006-02-07

Between 750,000 and 1 million people in the United States are addicted to heroin, a semisynthetic opioid made from the seeds of opium poppies. This highly addictive, illegal drug is converted in the brain into morphine, which binds to opioid receptors (which normally bind pain-relieving peptides produced by the brain) to produce a euphoric rush or heroin “high.” Repeated heroin use causes drug dependency—increasing amounts of the drug are needed to achieve its pleasurable effects, and its removal rapidly produces unpleasant withdrawal symptoms (“lows”) that can last for several days to months. Users become addicts when their desire to take heroin outweighs the negative health, social, financial, and legal consequences of their drug habit.

For more than 30 years, the synthetic narcotic (a drug that induces sleep) methadone has been used to treat heroin addiction. Methadone, a powerful pain-relieving drug, binds to the same receptors as heroin but without producing the euphoric rush. Because it lasts much longer in the body than heroin, patients trying to abstain from heroin need to take only a single daily dose of methadone to avoid withdrawal symptoms. Although patients become physically dependent on methadone, the reduction in withdrawal symptoms, together with a reduction in drug cravings, helps heroin addicts in methadone maintenance treatment programs stop using illicit drugs and lead normal lives.

The minimum maintenance dose of methadone recommended in these programs—60mg/day—is derived from randomized trials that have tested the ability of different doses of methadone to wean populations of addicts off heroin. However, many clinicians report that lower doses of methadone are effective in some patients. To test whether there is scientific evidence for these anecdotal observations, Jodie Trafton, Jared Minkel, and Keith Humphreys undertook a prospective observational study of individual responses to methadone treatment. They now report that setting a standard dose will not optimize therapy for all patients, and recommend that methadone doses be titrated on an individual basis to achieve heroin abstinence.

The researchers studied 222 volunteers addicted to heroin for a year after they started methadone treatment at eight clinic sites. Four of these sites regularly dosed patients with more than the recommended minimum daily methadone dose, and four dosed a significant number of patients with lower doses. Overall, 168 volunteers achieved heroin abstinence for at least a month, as measured by the absence of illicit opioids in their urine. The median effective daily dose of methadone taken by these successful volunteers was 69 mg, but doses ranged from 1.5 to 191.2 mg. Of those who abstained, 16% took daily doses of more 100 mg methadone, 38% remained abstinent on less than the recommended minimum daily dose, and almost half of the patients who did not achieve abstinence received more than 60 mg/day of methadone.

Trafton and colleagues also investigated the factors that might affect the methadone dose needed to achieve heroin abstinence. How long a patient had taken heroin and the amount taken per day did not correlate with the methadone dose associated with abstinence. However, patients who had previously been through drug detoxification treatments appeared to need higher methadone doses, as did those recently diagnosed with depression or posttraumatic stress disorder and those living in areas with lower average heroin purity. In addition, patients who were abstinent on higher doses were more likely to have stayed in treatment longer or attended a clinic where dose reductions were discouraged. Taken together, these factors predicted 40% of the variance in methadone dosage associated with heroin abstinence. The results suggest that only patients with lower methadone needs achieve abstinence in the early titration phase of treatment or at clinics that encourage use of lower doses.

These results provide scientific confirmation that the dose of methadone required to achieve heroin abstinence varies greatly between patients, and indicate that effective and ineffective dose ranges overlap substantially. The researchers suggest that clinicians should be allowed some flexibility in determining methadone dosing and call for research into the most effective way to determine the optimal dose for a particular patient. For now, they suggest, given that patients attending clinics that routinely give at least the recommended minimum dose of methadone do better on average than those attending clinics where lower doses are often given—60 mg/day should be the benchmark for dose titration, which should occur early during treatment.

## 

**Figure:**